# Clinical Feasibility of Applying Immersive Virtual Reality during Robot-Assisted Gait Training for Individuals with Neurological Diseases: A Pilot Study

**DOI:** 10.3390/brainsci14101002

**Published:** 2024-10-02

**Authors:** Daniele Munari, Angela von Wartburg, Veronica G. Garcia-Marti, Matjaž Zadravec, Zlatko Matjačić, Jan F. Veneman

**Affiliations:** 1 Hocoma Medical GmbH, 8604 Volketswil, Switzerland; danielemunari@gmail.com (D.M.); angela.vonwartburg@dih.com (A.v.W.); veronica.garcia@dih.com (V.G.G.-M.); 2 Research and Development Unit, University Rehabilitation Institute Republic of Slovenia, 1000 Ljubljana, Slovenia; matjaz.zadravec@ir-rs.si (M.Z.); zlatko.matjacic@ir-rs.si (Z.M.)

**Keywords:** virtual reality, medical robotics, neurology, neurorehabilitation, medical treatment

## Abstract

Background: Immersive virtual reality has the potential to motivate and challenge patients who need and want to relearn movements in the process of neurorehabilitation. Objective: The aim of this study was to evaluate the feasibility and user acceptance of an innovative immersive virtual reality system (head-mounted display) used in combination with robot-assisted gait training in subjects suffering from neurological diseases. Methods: Fifteen participants suffering from cerebrovascular accident or spinal cord injury completed a single session of immersive virtual reality using a head-mounted display during a Lokomat^®^ gait session. Training parameters and safety indicators were collected, and acceptance was investigated among participants and therapists. Results: The results suggest that an immersive virtual reality system is feasible in terms of safety and tolerance. Furthermore, the very positive overall acceptance of the system suggests that it has the potential to be included in a robot-assisted gait training session using Lokomat^®^. Conclusion: Overall, this study demonstrates that a fully immersive virtual reality system based on a head-mounted display is both feasible and well received by cerebrovascular accident and spinal cord injury patients and their therapists during robot-assisted gait training. This study suggests that such a virtual reality system could be a viable alternative to the screen-based training games currently used in neurorehabilitation. It may be especially suitable for enhancing patient motivation and adherence to training, particularly if the application is enjoyable and not mentally taxing.

## 1. Introduction

The recovery of functional gait is of great importance for neurological patients and is a key goal of their rehabilitation programs [1]. In recent years, the field of neurorehabilitation has shown a growing interest in advanced technologies, such as robotics and virtual reality (VR), due to their multipurpose application to patients’ recovery pathways [1,2,3]. For example, robot-assisted gait training (RAGT) is a safe form of treadmill training that reduces physical demands on therapists and allows highly repetitive gait training early in the rehabilitation process [4,5]. In RAGT, depending on the applied technology, different training parameters, such as treadmill speed, amount of body weight support, or amount of support for leg motion, can be adjusted to create an optimized training intensity [6]. Notably, systematic reviews and meta-analyses have reported meaningful effects in different acute and chronic neurological conditions [7], including stroke [8,9], traumatic brain injury [10], spinal cord injury (SCI) [11], multiple sclerosis [12], and Parkinson’s disease [13], corroborating the added value of integrating robotic technologies into standard rehabilitation programs across clinical populations. Feedback and gamification are used to enhance the engagement and motivation of patients undergoing such training [14,15]. Currently, such “games” are usually based on tasks executed on a computer screen; they are thus partially disconnected from everyday gestures and movements and are therefore different from the natural experience of walking in an environment [16]. In contrast, task-specific and context-specific training conditions have been proven to be key features for the optimal transfer of acquired motor skills to real life [17].

Researchers have shown that fully immersive VR-based training can be an effective tool for both assessing and aiding motor and cognitive rehabilitation [14,18,19]. VR is defined by three primary elements: immersion, interactivity, and sense of presence. Immersion measures how fully a virtual environment (VE) can deliver multisensory feedback coordinated with user interaction. Interactivity defines how able the user is to manipulate their environment. The sense of presence comes from the fusion of those two, measured by how much real-world sensations are countered by the VR experience. VR systems are classified as fully immersive, semi-immersive, or non-immersive. Fully immersive VR tools include head-mounted displays (HMDs) and cave automatic virtual environments (CAVEs), while semi-immersive tools use large screens or projectors for moderate immersion, and non-immersive devices range from PCs with standard monitors to tablets. The application of various VEs applying different levels of immersion has allowed VR to offer diverse stimulation in the context of movement training and has improved opportunities for repetitive tasks, feedback provision, and sustained motivation, enhancing sensory inputs, and facilitating motor learning [20,21]. Additionally, it has been suggested that immersive VR promotes deeper engagement, which can lead to synaptic changes at the cortical and subcortical levels, which are crucial for motor relearning [22].

A fundamental aspect that needs to be considered while developing a VE system is the possibility of “cybersickness,” which may to some degree be unavoidable in the use of VR [23]. When visual response processing for user input interaction is delayed, the signals from the eyes, the vestibular system of the inner ear, and proprioceptive receptors to the brain conflict with each other, and motion sickness may be triggered [24]. Therefore, the user’s experience of cybersickness must be reduced according to the influencing factors of cybersickness [25]: (1) the user actively can control and adjust their viewpoint; (2) avoid or limit linear or angular acceleration or deceleration to reduce stimulation of the vestibular organs; (3) display visual indicators or movement tracks to users; and (4) dynamically blur the unimportant areas in the field of view.

While fully immersive VR presents a novel method to engage and motivate patients during RAGT, experimental evidence of its feasibility and effectiveness remains limited [26,27]. To the best of our knowledge, among the few studies that have investigated the general feasibility in the clinical setting and acceptance of a fully immersive VR system in neurological patients, no study has focused on the feasibility of using an HMD in synchronization with an RAGT device. Thus, the primary aim of this study was to investigate the feasibility of using, in a real clinical treatment context, an innovative, fully immersive VR system (applying an HMD) during RAGT for subjects suffering from neurological disorders. The secondary aim was to assess the acceptance of this approach in a clinical environment by collecting patients’ opinions on the experimental configuration as well as those of the treating therapist.

We first hypothesized that fully immersive VR during RAGT is a practically feasible treatment option in a clinical setting for subjects suffering from neurological disorders. Additionally, we expected that the innovative integration approach would find positive acceptance among both participants and therapists.

## 2. Materials and Methods

### 2.1. Study Design and Setting

This feasibility study was conducted between January 2022 and July 2022. All study participants were recruited at the University Rehabilitation Institute, Republic of Slovenia.

The study was approved by the Slovenian National Medical Ethics Committee (0120-548/2021/3) and was carried out in accordance with the tenets of the Helsinki Declaration. All participants gave their informed written consent to participate in the study.

### 2.2. Participants and Recruitment

For the type of feasibility study presented in this paper, the recruitment of 15 participants was considered adequate, as this would provide enough data to assess the practicality and safety of the treatment approach. This sample size allows the identification of common issues and variability in patient response while fitting within the typical range for feasibility studies [28]. This study is explicitly not intended to measure the treatment’s effects on the investigated configuration.

Subjects suffering from cerebrovascular accidents (CVAs) or SCIs were included in this study. All subjects received three, four, or five RAGT sessions per week over a period of three weeks, during which they were invited to test the fully immersive VR system in a single session (in combination with the RAGT). Before starting the VR session, participants had already participated in at least three Lokomat^®^ training sessions, and thus were already familiar with the RAGT setup. The inclusion criteria were as follows: (1) age between 20 and 60 years; (2) ability to move lower extremities; (3) partial or impaired ability to walk with or without medical walking aids; (4) ability to participate in the study; and (5) cognitive abilities to understand and follow simple verbal instructions. In addition, the following Lokomat^®^-specific inclusion criteria were also considered: (1) body weight < 130 kg; (2) body height < 200 cm; (3) no other associated neurological dysfunction, such as reduced sensation, contractures, or cardiovascular diseases. Exclusion criteria were as follows: (1) considerably reduced bone density (osteopenia or osteoporosis); (2) leg length difference < 2 cm; (3) non-consolidated fractures; (4) restricted range of motion in lower extremities; (5) excessive spasticity or contractures of lower extremities; (6) presence of disabling sensory alterations, including hallucinations; and (7) concomitant medical and psychiatric illness possibly interfering with the robotic/VR training. These conditions were judged for the individual patients by the therapist supervising the study.

The therapists involved in the study selected 15 patients (diagnosed with either SCI or CVA) among those already receiving Lokomat^®^ treatment in accordance with the above-mentioned criteria.

These 15 included subjects were characterized by the following statistics: months after diagnosis: 9.2 ± 11.21, age: 54.3 ± 11.97 years, height: 177.7 ± 9.97 cm, weight: 84.8 ± 16.49 kg, and FAC: 3.5 ± 1.46. Five of the participants were diagnosed with SCI. Seven were diagnosed with the right body side affected by CVA. Three were diagnosed with the left body side affected by CVA. Table 1 shows the demographic and clinical characteristics of the participants.

Although not monitored in the study, it is relevant to note that around 20% of the subjects with CVA and around 50% of the subjects with SCI who were already included for Lokomat^®^ treatment were invited to participate in the study by their responsible therapist. The differences between the CVA and SCI groups were related to the occurrence of cognitive and perceptual limitations in the CVA population.

### 2.3. Experimental Apparatus and Control

#### 2.3.1. Robot-Assisted Gait Training Device

**Figure 1:**
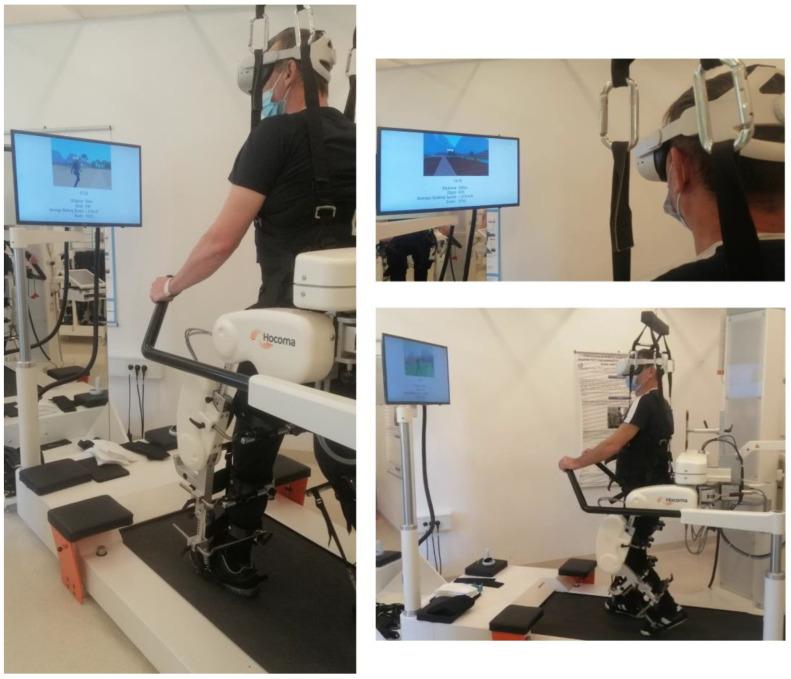
Patient walking in the Lokomat^®^ Pro System version 6, wearing a head-mounted display (Meta Quest 2) to visualize the immersive virtual reality.

The RAGT device used in this study was the Lokomat^®^ Pro V6 FreeD (Hocoma AG, Volketswil, Switzerland). This device is commonly used to treat selected neurological patients in the clinic where this study was performed (the University Rehabilitation Institute Republic of Slovenia, Ljubljana, Slovenia). The Lokomat^®^ is a treadmill-based robotic leg orthosis–gait training device with actuated hip and knee joints and a dynamic body weight support system that supports the patient using a harness [29] (see Figure 1). The orthosis is programmed to follow a predefined gait pattern with an impedance control strategy that allows adjustment of the rigidity/stiffness. In standard therapy, the gait pattern and the mechanical impedance of the robotic hip and knee joints (called “guidance force” in the Lokomat^®^ software version 6.5) are manually adjusted by the therapist through the user interface. The guidance force is constant throughout the gait cycle.

#### 2.3.2. Immersive Virtual Application for Use during RAGT

An immersive VR application was developed with the Unity game engine (Unity Technologies, San Francisco, CA, USA) and the plugin FMETP STREAM (Frozen Mist), which allows live video streaming and cross-platform network communication. The Unity game engine was selected as adequate for VR application development, as it was already commonly used by the development team in Hocoma. The Meta Quest 2 HMD (Oculus by Facebook, Inc.—Meta Technologies, LLC, Menlo Park, CA, USA [30]) was used as the hardware platform to operate the application. This device was selected in line with the argument that it is considered suitable in the context of neurorehabilitation, considering its level of performance and physical characteristics (weight, center of mass) [16].

**Figure 2:**
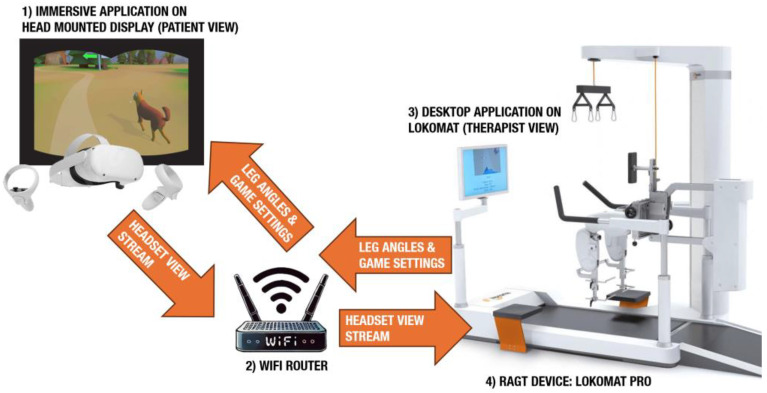
Representation of the architecture of the integration of an immersive VR application through an HMD (**1**) into a commercial RAGT device (**4**), which mutually communicate the indicated information through a WIFI network (**2**). The VR application receives setting and real-time leg angles from the RAGT device, and the RAGT device receives the real-time visual stream from the HMD that is displayed in a desktop application on the patient screen (**3**). In this context, the patient screen is used to inform the therapist of the actual view of the patient in the VR scene.

The combination with the RAGT device was set up as follows: a wireless connection was established between the VR headset and a router connected to the Lokomat^®^ with a wired connection. Through this connection, the headset and the Lokomat^®^ exchange measured signals, parameters, and video streams. See Figure 2 for an architectural view of the system.

**Figure 3:**
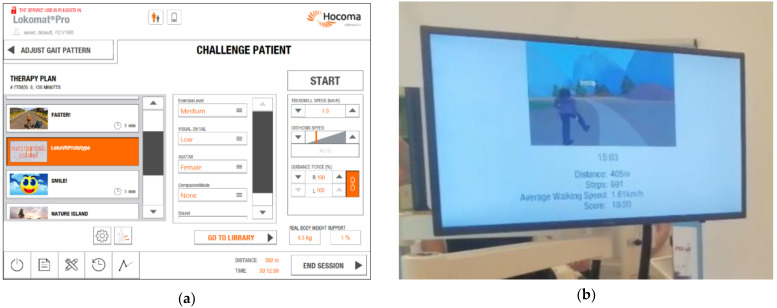
(**a**) Integration of the fully immersive VR system in the Lokomat^®^ therapist interface (LokoControl); (**b**) Patient’s screen of the Lokomat^®^ (this screen is used to present content to the patient during normal Lokomat^®^ operation; however, in this study, the screen was used to display the view of the patient in the VE to the therapist, along with additional training data).

The operator control of the VR add-on was integrated into the Lokomat^®^ therapist pnterface (LokoControl) in a style similar to that of other therapist control functions (see Figure 3a). The Lokomat^®^ screen, normally used to present content to the patient, was used in this study to stream the VE displayed in the HMD in real time (see Figure 3b) so that the therapist could see what the patient saw and looked at, including the hands of the patient if in view. This supports the therapist in providing adequate information and feedback to the patient during the training session. In addition, instructions and performance data were displayed to the therapist on this screen (see Figure 3b).

As the feasibility of VR is related not only to the hardware but also to the details of the application in use, a detailed description and a video impression are provided here. The VR environment consists of an island with a lighthouse, trees, green grass, mountains, bridges, and some landmarks, all in low-poly stylized graphics. Matching ambient sounds of twittering birds and wind create a relaxing natural atmosphere. Multiple paths lead to different areas, allowing exploration of the whole island, although movement is limited to predefined paths.

**Figure 4:**
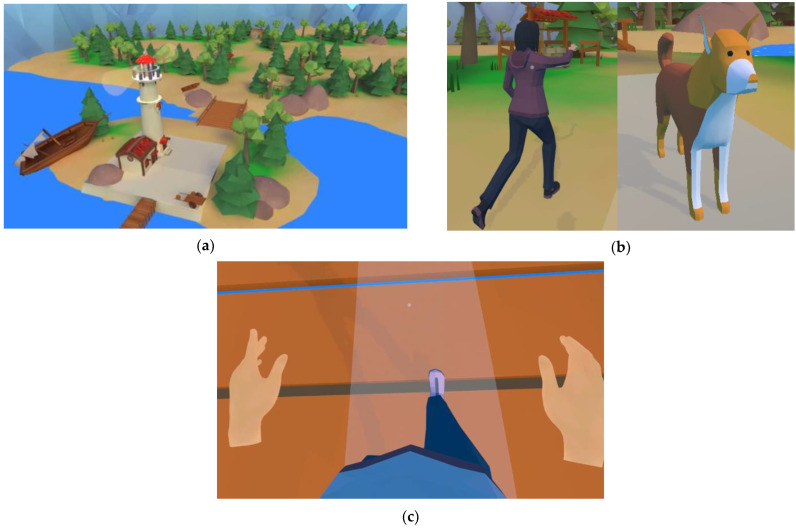
(**a**) Lighthouse park environment, (**b**) human and dog companions, and (**c**) avatar displaying the patient’s leg and hand postures and movements in the VR from a first-person perspective.

**Figure 5:**
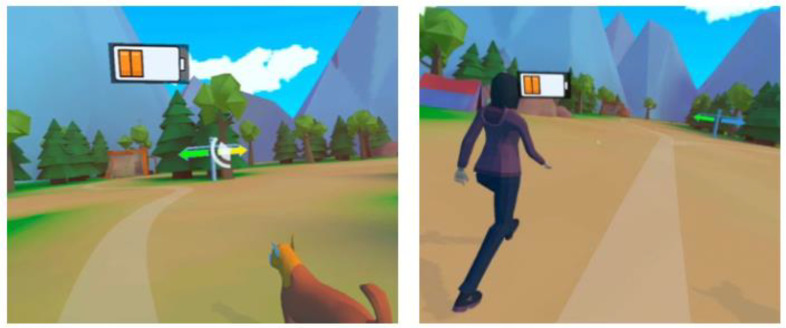
Representative 3D screen captures of the virtual environment with companions and the battery collection.

The patient is represented in virtual reality by an avatar human body (see Figure 4c). However, instead of arms, only hand representations are visible; arms are missing from the shoulders down. The patient’s hand movements are tracked by the Meta Quest 2′s hand-tracking capabilities and applied to the virtual hands. The orthosis’s leg angles, measured by the Lokomat^®^, are sent to the headset through the wireless network connection and mapped to the legs of the patient’s avatar. The presence of an avatar body with legs, and the first-person view that places the camera at the head position of the virtual avatar, let the patient see their leg movements on the avatar when looking down, in a naturalistic way. This creates the illusion that they are walking in the virtual environment by themselves and that they are seeing their own legs and their own movements. The avatar’s leg movements are used to generate the forward motion of the avatar on the predefined paths in the virtual world. At junctions, the desired path can be chosen by looking at the signpost pointing in the preferred direction. For pointing, the orientation of the headset is used; there is no eye tracking. The mechanism of looking at something for selection is further used to collect objects along the path, which is one of the two possible tasks the therapist can choose for the patient (see Figure 5). The other task uses the patient activity, a metric calculated by the Lokomat^®^ based on its force sensor readings that reflects the effort contributed to the orthosis movement by the patient. For a given consistently high value of patient activity, the virtual environment changes its coloring to brighter, more saturated colors, adds flowers to the path in front of the avatar, and adds layers of music. With decreased patient activity over time, these rewarding additions are slowly removed. While exploring the island, the patient’s avatar can be accompanied by one or two social companions (See Figure 4).

The therapist can select companions in the LokoControl user interface with the option to display none, one, or both companions. The companion options consist of a female human or a dog. The human companion’s leg movements are synchronized with the Lokomat’^®^s orthosis movement and therefore also with the patient’s and the avatar’s leg movements (idea originating from [31]). To further mitigate the risk of motion sickness (cybersickness) associated with navigating the default curved paths, there is the possibility to traverse a straight path across the island. This setting can be selected by the therapist as an introductory option when introducing immersive virtual reality to new users or for individuals who are more susceptible to motion sickness. An impression of the VR application can be obtained through the video recording that is provided as supplementary material to this paper (Video S1).

### 2.4. Treatment Procedures

The subjects tested the fully immersive VR system in a single session. During a Lokomat^®^ gait training session, the participants wore the HMD (see Figure 1) to immerse them in the VE through the application, as described in Section 2.3.2 (see Figure 4). The Lokomat^®^ session lasted between 30 and 45 min, with intermediate rests as tolerated by the patient and decided by the responsible therapist. Walking speed progressively increased to a comfortable level. The sessions were provided by qualified therapists who operate the Lokomat^®^ system daily. System safety was ensured through established Lokomat^®^ safety mechanisms, and the wearing of an HMD was expected to have minimal impact on safety. Additionally, sessions were closely monitored by a technically trained observer who could stop the device if any risks or dangers were detected. Also, an emergency stop button is always within reach of the therapist, according to the design of the Lokomat^®^ for normal operation.

### 2.5. Assessment Procedures

#### 2.5.1. Sociodemographic and Clinical Variables

A physician completed a report documenting the clinical features of the participants, including type of neurological disorder (cerebral vascular accident or spinal cord injury), affected side, onset of illness, age, height, weight, gender, FAC score [32], and mini-mental state examination (MMSE) score [33]. After the session, the participant and the therapist each filled out a targeted questionnaire indicating their acceptance of the session.

#### 2.5.2. Feasibility and Session Adherence in the Clinical Setting

The clinical feasibility of the setting was assessed by collecting the following training parameters during the sessions: level of the guidance force (GF) (%left/%right—a standard Lokomat^®^ setting on the amount of provided gait support), walking speed (km/h), body weight support (BWS) (% of body weight), and fully immersive VR session duration (min). The safety indicators were any occurrence of side effects or adverse events, such as pain, motion sickness, dizziness, exertion, fatigue, and headaches, as well as the number of withdrawals (n). Common Terminology Criteria for Adverse Events (CTCAE) version 3.0 was used to assess the adverse effects of exercise intervention [34].

#### 2.5.3. Acceptance

Based on the established inclusion criteria, the therapists responsible for patient treatment initially selected the Lokomat^®^ patients they deemed suitable for HMD training. We determined prospective acceptability by the proportion of patients approached who accepted an offer to participate in the “fully immersive VR” session [28,35]. Reasons for declining the intervention were collected.

Acceptance was also investigated by means of questionnaires developed by the authors based on the technology acceptance model (TAM) [36], one for the participants and one for the therapists. The first, the patient questionnaire, consisted of 18 closed-ended questions, which the patients had to answer by means of a 5-point Likert scale (1: strongly disagree; 5: strongly agree) [37] and four open-ended questions (see Table 2). The validated TAM questionnaire was modified for this research to consider the specific situation in which a therapist prescribes the technology for a patient under treatment. The questions of the TAM are not directly applicable to such a configuration and were therefore modified to fit the use case of this study. The full questionnaire used is provided.

The second, therapist questionnaire, consisted of 11 closed-ended questions where the therapists had to respond by means of a 5-point Likert scale (1: strongly Disagree; 5: Strongly Agree) [37] and 2 open-ended questions (see Table 3).

### 2.6. Data Collection and Management

Data were ordered and collected in an electronic sheet and stored securely in a database at the rehabilitation center. For the feasibility measures, descriptive statistics for the level of the Lokomat^®^ guidance force, walking speed, body weight support, and fully immersive VR session duration were reported and analyzed descriptively (mean value, standard deviation). For safety indicators, any occurrences of pain, fatigue, dizziness, or possible falls were reported. The number of dropouts was also documented. Questionnaire data were processed, and the results were analyzed descriptively (median, 25/75% percentiles and outliers).

## 3. Results

In the following section, we report the results of the feasibility study of the fully immersive HMD-based VR system during RAGT that we performed in a cohort of 15 patients. The evaluation was made with each patient after performing their single session.

### 3.1. Feasibility and Session Adherence

As reported in Table 4, all 15 subjects performed the training session according to personalized settings (GF: 96 ± 7.37, walking speed: 1.40 ± 0.19 BWS: 43.3 ± 7.83, and fully immersive VR session duration: 20.9 ± 6.76); one of the participants had to prematurely stop the session. No musculoskeletal events of CTCAE version 3.0 > Grade 2 occurred, and no significant adverse events or side effects were reported during any session.

### 3.2. Acceptance

**Figure 6:**
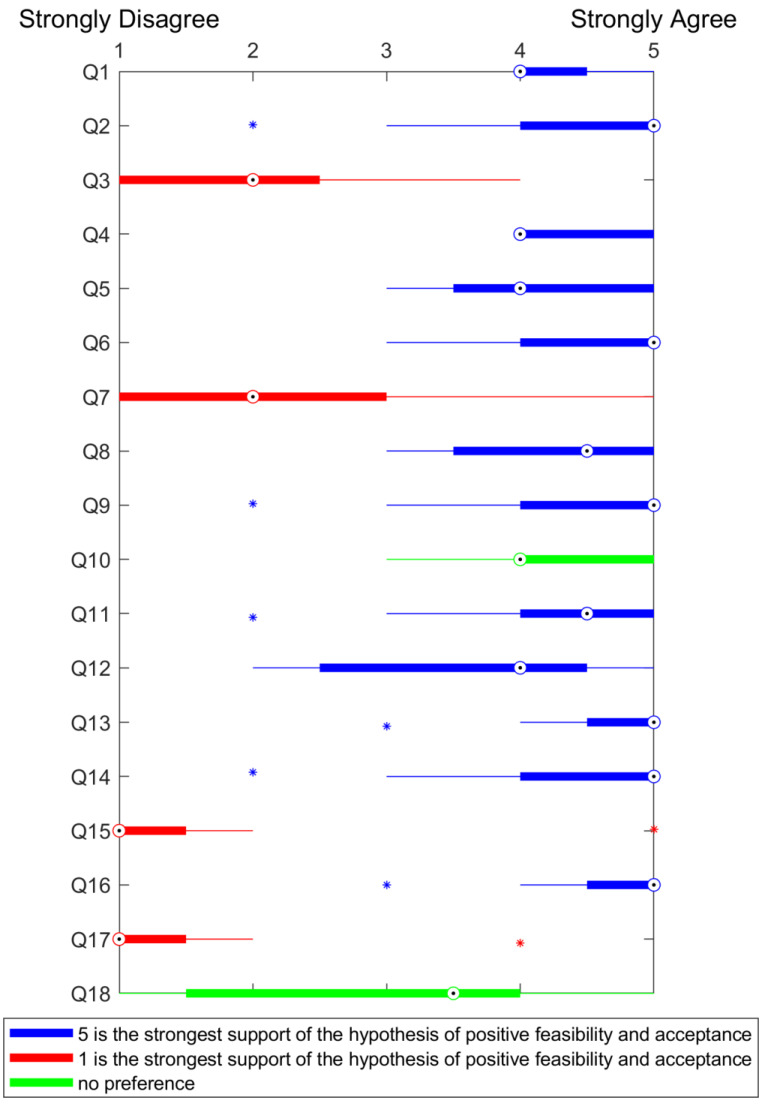
Boxplot displaying the patients’ questionnaire scores, with the central circled dot representing the median. The left and right edges of the box (solid line section) correspond to the 25th and 75th percentiles, respectively. The whiskers extend to the furthest data points, while outliers are marked separately with asterisks.

**Figure 7:**
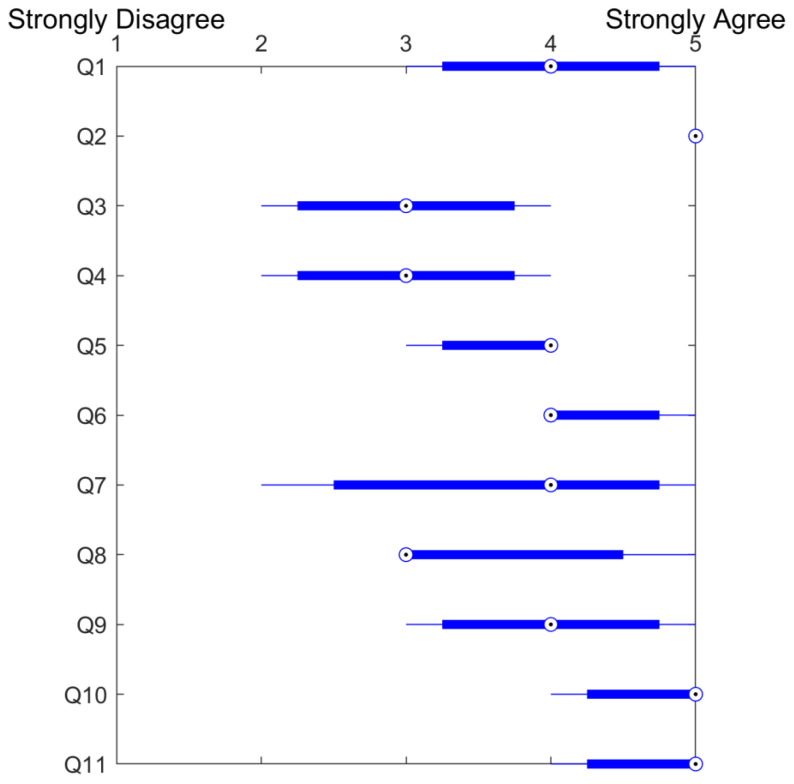
Boxplot displaying the therapists’ questionnaire scores, with the circled dot representing the median. The left and right edges of the box (solid line section) correspond to the 25th and 75th percentiles, respectively. The whiskers extend to the furthest data points.

Fifteen of the invited participants accepted the intervention and fourteen concluded the fully immersive VR session during Lokomat^®^ Pro training, following the judgment of each patient’s therapist. One subject with CVA exhibited concentration disorders, neglect, and hemianopsia, which hindered proper cooperation with the VR game, leading to premature termination of the session. The results of the questionnaires are summarized in Figure 6 and Figure 7.

Open-ended replies provided by the participants after the session further confirmed that the fully immersive VR session was considered acceptable (Table 5). All participants liked the virtual environment: “like the environment, nature, which relaxes”; “it felt positive”. Participants enjoyed walking with a companion and expressed willingness to be more challenged: “to go uphill to mountain hiking”; “there could be some walking obstacles in the walking path”. Several participants also provided valuable insights for future iterations of immersive VR application design.

Open-ended replies provided by the therapists after the session also confirmed that the fully immersive VR session was considered acceptable and usable (Table 6). Therapists noted that the approach was easy to use, “the whole installation is fairly easy to use”, and participants were motivated, “the patients had more motivation for training—especially those, who had several Lokomat^®^ trainings before participation”. Furthermore, the therapists provided valuable insights for future iterations of fully immersive VR application design.

### 3.3. Further Observations

The therapists reported that they consistently offered patients the opportunity to take breaks between each ‘virtual walk,’ which lasted around 10 min. However, patients always preferred to continue, often completing up to three walks consecutively.

Although this was anticipated by some therapists, none of the patients or other users complained about neck or back pain during or following the sessions as an effect of the use of the HMD.

## 4. Discussion

The results of this feasibility study suggest that using an innovative, fully immersive, HMD-based VR system displaying the developed VR application in combination with RAGT is feasible for subjects suffering from neurological disorders, with respect to safety, adherence, and tolerance, within certain inclusion criteria. Furthermore, the highly positive overall acceptance of the system among both participants and therapists suggests that the immersive VR system may add motivational value as an optional feature in a regular Lokomat^®^ Pro session, to the potential benefit of patients. Taken together, our results motivate further studies to explore and validate the clinical efficacy of such a combination or extension of regular Lokomat^®^ training. As with all pilot studies with a limited sample size, these results should be interpreted with caution and may not be generalizable to the CVA and SCI populations at large.

The novelty of this study is the implementation of an innovative, fully immersive VR environment integrated and synchronized with a RAGT session. The successful application of a fully immersive VR system may lay the groundwork for additional treatment options for neurorehabilitation [19,38,39]. Fully immersive VR platforms, particularly HMDs, provide a rich experience and credibility that cannot be achieved by semi-immersive VR modalities. They also enable the provision of treatment in alternative environments that aid relaxation, more closely approximate the real environment, or build safety awareness without jeopardizing patient safety. Fully immersive VR also provides the unique benefit of occluding the hospital or clinic environment, thus minimizing auditory and visual distractions while highlighting preferred stimuli (in this case, of the affected extremity) [40]. It should be stressed that fully immersive VR has several requirements for motor and cognitive rehabilitation interventions: repetitive practice, feedback about performance, multimodal stimulation, and controlled, safe, and ecologically valid environments [41].

Over the past decade, technology-based exercise interventions have gained wide interest worldwide. VR, both immersive and non-immersive, has become a mature technology and is currently experiencing increasing adoption for clinical research, psychological interventions, and cognitive studies [19,42,43,44,45]. Specifically, the use of VR as a physical therapy tool in neurorehabilitation was recently the subject of analysis in a systematic review, and the conclusion from the authors was that VR induced changes in neural plasticity for survivors of stroke and optimized functional recovery [46]. In addition, the authors highlighted that, compared with regular computer monitors, participants in the immersive VR system reported more presence and a better learning experience. For instance, researchers have recently reported multiple cases of consistent neurological recovery in people with SCI following a protocol that integrates locomotion training with other systems such as brain–machine interfaces (BMI) [47,48,49].

### 4.1. Feasibility

The main objective of this study was to evaluate the clinical feasibility of a novel immersive VR integration approach during RAGT for individuals with neurological disorders. Participants demonstrated 93% adherence to the protocol, surpassing results from other studies on VR feasibility [45,50]. In the study by Song et al., two subjects did not complete the intervention because of early discharge from the hospital [45]. In another study by Fowler et al., 10 of 14 participants (71%) who participated completed the VR session [50]. Therefore, it is important to note that therapists preselected participants based on specific inclusion criteria, which may directly influence the level of adherence.

No adverse effects were reported during the VR sessions, affirming the safety of the system and the intervention method. One participant discontinued due to cognitive impairments based on the judgment of the therapist; this was not related to the safety of the patient. Many clinical trials have shied away from using head-mounted displays and immersive VR due to concerns over safety and cybersickness [25]. This study’s fully immersive VR application was developed with these issues in mind, using a commercial HMD. This must therefore be considered an essential factor underlying feasibility. Consumer-grade, fully immersive VR technologies have advanced considerably over the past five years [37]. These affordable technologies could be highly beneficial in real clinical environments [47,50,51,52].

### 4.2. Acceptance

The secondary objective was to evaluate the approach’s acceptance by participants and therapists within a clinical setting. The acceptance of immersive VR in neurorehabilitation is a critical issue that has not been extensively explored.

Overall, the fully immersive VR session during RAGT was widely accepted. Most participants found the intervention suitable for their needs and expressed a willingness to undergo it again in the future. Many participants described the fully immersive VR experience as positive and enjoyable. Participants reported high scores in terms of the enjoyability, challenge, and effectiveness of the immersive VR experience. Common reasons for enjoying the experience included the sense of immersion, the physical activity, and the novelty of the experience. In this way, immersive VR may address patients’ unmet needs to “escape” the hospital environment and experience some degree of normalcy during training.

Therapists also found the fully immersive VR session acceptable and usable. They regarded the integration of the VR system during RAGT as a valid and reliable tool for enhancing participants’ engagement. These qualitative findings were further bolstered by most participants’ endorsement that they would use VR again if given the opportunity. Additionally, the qualitative results identified areas for usability improvements. Participants provided feedback that could guide future iterations, such as incorporating more variations in immersive VR tasks to maintain motivation during RAGT.

### 4.3. Future Directions

This study demonstrated the feasibility of employing a fully immersive HMD-based VR system during RAGT and its acceptance among participants with neurological disorders and their therapists. Our feasibility study produced promising outcomes for utilizing VR in gait rehabilitation.

Properly designed VR applications enable active patient engagement in treatment and promote a patient-centered approach that considers relative aspects, such as the severity of the neurological condition and cognitive function. While VR cannot replace real experiences and task-specific training, it may enhance the acceptance of treatment. Emerging evidence indicates that a combination of physical and cognitive factors is crucial for recovery after neurological disorders [26,47,48,49]. For instance, VR has been shown to be a potentially valuable tool in motor assessment and rehabilitation during motor rehabilitation [14,53]. VR-based training offers increased opportunities for repetitive practice, feedback, and motivation for sustained practice by applying visual, auditory, and tactile inputs to enhance motor learning; however, potential risks, such as the specific or long-term effects of this training modality on motor and cognitive aspects, need further examination and cannot be inferred from a short-term feasibility study.

There is a need for high-quality randomized controlled trials (RCTs) with larger sample sizes and standardized outcome measures for common functional outcomes to improve the generalizability of the results and better understand the impact on treatment outcomes. Furthermore, risk assessment and analysis could be conducted to evaluate whether the benefits of immersive VR outweigh the risks of using an HMD in a specific context and implementation.

### 4.4. Limitations

While this study presents innovative and valuable findings, it is important to consider several limitations when interpreting the results.

First, as a feasibility study, there was no control group against which to compare these findings. Furthermore, the sample size was small. Randomized controlled trials involving a larger sample could generate stronger evidence on the acceptance of immersive VR for individuals with neurological disorders and compare it with alternative training modalities, such as screen-based training games.

Second, the study relied solely on modified questionnaires (such as the TAM questionnaire) to gauge participants’ and therapists’ acceptance. In subsequent studies, especially once these technologies are integrated into clinical practice, quantitative methods could be employed to track changes in acceptance and adherence over time.

Third, the innovative immersive VR was tested in a single session. The long-term feasibility of the system should be assessed over multiple sessions.

Fourth, this paper primarily focuses on the feasibility of the approach without assessing its therapeutic effects. Moving forward, a rigorous randomized controlled trial should be designed to evaluate the efficacy of a fully immersive VR system during RAGT, paying close attention to the practical application details and the effects on long-term rehabilitation outcomes, such as motor function and quality of life.

## 5. Conclusions

This pilot study investigated the feasibility of an innovative, fully immersive, HMD-based VR system displaying a custom-made VR application in combination with RAGT for subjects suffering from CVA and SCI with respect to safety, adherence, and tolerance.

The positive overall acceptance of the system among participants and therapists suggests that the immersive VR system may add motivational value as an optional feature in a regular Lokomat^®^ Pro session, to the potential benefit of the patients. Furthermore, the results suggest that such a VR system could be a viable alternative to the screen-based training games currently used in neurorehabilitation. It may be especially suitable for enhancing patient motivation and adherence to training, particularly if the application is enjoyable and not mentally taxing. However, despite these positive findings, whether the benefits of immersive VR outweigh the risks of using an HMD should be evaluated on a case-by-case basis, considering the therapist’s judgment and the patient’s personal preferences, as well as be based on prospective evidence of the impact on the outcomes of such treatment modalities.

## Figures and Tables

**Table 1:** Demographic and clinical features of participants. For CVA-diagnosed patients, the mini-mental state examination (MMSE) was performed to assess their mental state. Participants suffering from SCI had no cognitive problems and therefore did not undergo the MMSE.

PID	Diagnosis	Affected Side	Onset (Months)	Age (Years)	Height (cm)	Weight (kg)	Gender (M/F)	FAC (Level)	Walking Ability	MMSE (Score)
1	SCI	both	48	33	162	60	F	1	Wheelchair	-
2	SCI	both	8	64	184	105	M	4	Wheelchair	-
3	CVA	right	5	54	185	93	M	5	Rollator	28/30
4	CVA	left	4	53	185	105	M	1	Wheelchair	27/30
5	CVA	right	4	57	185	81	M	1	Wheelchair	27/29
6	CVA	right	7	75	176	78	M	5	Rollator	27/30
7	CVA	left	12	64	173	80	M	4	Wheelchair	26/30
8	SCI	both	4	51	174	83	M	4	Wheelchair	-
9	CVA	right	5	46	194	108	M	5	Crutches	27/28
10	CVA	right	4	48	191	104	M	4	Wheelchair	28/30
11	SCI	both	4	65	168	88	M	3	Wheelchair	-
12	SCI	both	4	70	177	73	M	3	Crutches	-
13	CVA	left	5	52	171	69	M	3	Wheelchair	24/30
14	CVA	right	10	34	160	55	F	4	Walking stick	28/30
15	CVA	right	14	49	181	90	M	5	Walking stick	27/30
Mean			9.2	54.3	177.7	84.8		3.5		
(SD)			±11.21	±11.97	±9.97	±16.49		±1.46		

Abbreviations: FAC = functional ambulation category; PID = patient identification Number; SD = standard deviation; MMSE = mini-mental state examination.

**Table 2:** Acceptance questionnaire for the participants.

Number	Question Type	Question
Q1	Closed-ended	I think I can benefit from this technology
Q2	Closed-ended	The use of Virtual Reality makes Lokomat^®^ training more enjoyable to me
Q3	Closed-ended	The use of Virtual Reality makes me exercise harder in the Lokomat^®^
Q4	Closed-ended	The use of Virtual Reality adds challenge to the Lokomat^®^ training
Q5	Closed-ended	I think this way Lokomat^®^ training can be more effective for me
Q6	Closed-ended	I feel safe when using this technology
Q7	Closed-ended	Use of this technology can have negative consequences I can’t predict
Q8	Closed-ended	I feel confident when using the VR in the Lokomat^®^
Q9	Closed-ended	I feel like I can do what is required when using the VR in the Lokomat^®^
Q10	Closed-ended	I would rather have more things to do when walking in VR
Q11	Closed-ended	I like the environment I walked in
Q12	Closed-ended	I liked the companions that were walking with me
Q13	Closed-ended	It feels like time flies when training in the Lokomat^®^ with VR
Q14	Closed-ended	Wearing the VR headset is comfortable
Q15	Closed-ended	I felt sick when walking in the VR
Q16	Closed-ended	I felt comfortable during the training
Q17	Closed-ended	Looking at virtual reality makes me dizzy
Q18	Closed-ended	I forget I am in the clinic when training like this
Q19	Open-ended	What did you like or dislike about the virtual environment you walked in?
Q20	Open-ended	Which companion do you prefer to walk with, animal or human, and why?
Q21	Open-ended	Was there something that you would have liked to do in the virtual environment that wasn’t possible?
Q22	Open-ended	Do you have any good ideas for something you would like to do in a virtual reality when in the Lokomat^®^?

**Table 3:** Acceptance questionnaire for the therapists.

Number	Question Type	Question
Q1	Closed-ended	I felt confident operating the Lokomat^®^ together with the VR headset
Q2	Closed-ended	The amount of time spent donning/doffing the VR headset is feasible for use in clinical practice
Q3	Closed-ended	I am satisfied with the ease of operation of the device through the user interface
Q4	Closed-ended	I was able to adjust the game settings to address the unique needs of individual subjects
Q5	Closed-ended	Using the device did not interfere with my ability to provide appropriate supervision and guarding of the subject throughout all sessions
Q6	Closed-ended	I was able to communicate with the patient during the training as needed
Q7	Closed-ended	VR headset was compatible with gait training activities
Q8	Closed-ended	I felt that VR had a positive impact on the subjects’ walking performance
Q9	Closed-ended	I felt that the patient(s) enjoyed the VR training session
Q10	Closed-ended	Using the Lokomat^®^ together with a VR headset would be useful in my clinical practice
Q11	Closed-ended	I would recommend using the Lokomat^®^ together with a VR headset to other physiotherapists
Q12	Open-ended	Please note 3 things you liked about using the Lokomat^®^ together with a VR headset
Q13	Open-ended	Please note 3 things about using the Lokomat^®^ together with a VR headset you would like to change

**Table 4:** Lokomat^®^ session and immersive VR parameters.

PID	GF Left/Right (%)	Walking Speed (km/h)	BWS (%)	Immersive VR Session Duration (min)
1	90/90	1.50	30	32
2	80/80	1.70	40	22
3	100/100	1.70	44	27
4	100/100	1.30	45	10
5	100/100	1.50	43	23
6	100/100	1.30	50	16
7	100/100	1.20	50	13
8	80/80	1.60	30	20
9	100/100	1.40	49	11
10	100/100	1.50	48	20
11	100/100	1.60	28	18
12	90/90	1.70	45	31
13	100/100	1.20	49	19
14	100/100	1.20	50	28
15	100/100	1.30	49	23
Mean	96	1.4	43.3	20.9
(SD)	±7.37	±0.19	±7.83	±6.76

Abbreviations: BWS = body weight support; GF = guidance force; VR = virtual reality; SD = standard deviation; PID = patient identification number.

**Table 5:** Open-ended replies from the participants. Direct quotations from patients are indicated with quotation marks; the other comments are observations by therapists.

Q19—What did you like or dislike about the virtual environment you walked in?
- “I like the environment, nature, which relaxes” - “nature is ok, lady is awful, dog is nice” - “it felt positive” - “after we explain what to do in the VR, it made sense to the patient and he liked it” (observation) - “the patient liked the dog barking” (observation) - “the walk, nature, mountains” - “nice scene”; the patient fully trusts the VR’s designers to create the best VR for the treatment/training
Q20—Which companion do you prefer to walk with, animal or human, and why?
- half of the patients chose the animal - three patients voted for both companions - one would choose to walk with the lady - one would choose the animal because the low graphic resolution meant the lady was not pretty
Q21—Was there something that you would have liked to do in the virtual environment that wasn’t possible?
- “I would like to enter the buildings (lighthouse, tent), where there could be some additional tasks to do“ - “to go uphill to mountain hiking” - “I would like to swim at the end” - “there could be some walking obstacles in the walking path” - “I would like to gather mushrooms” - “I would like to see the birds, since they are tweeting”
Q22—Do you have any good ideas for something you would like to do in a virtual reality when in the Lokomat^®^?
- “I would like to have more activities, e.g., birds or other animals beside batteries” - “jump into the water at the end of the walking path” - “there could be two variants of batteries: those, which gives you power (green batteries) and those, which decrease power (red batteries)” - “I would like to open the box, which can be done by using virtual hands mode in VR; by the end of training I felt a bit bored due to the same type of game (just collecting the batteries)” - “I get bored because it’s always the same game” - “I would like to throw the bone for the dog” - “the game could be gradually more challenging” - “are the batteries always on the same locations?” (yes) - “the batteries could appear when being in a predefined radius, because at the current state of the VR, the very distant batteries are hard to collect and the patient is trying to collect them” - “the counter for batteries could be added” - “batteries are not really in the context of nature—consider replacing batteries with something that suits in nature, e.g., mushrooms or similar natural objects” - “I would like more people or animals walking around in the park and they would not be necessary the walking companions” - “I would be satisfied if there could be more things to do in VR” - “I was really satisfied with the VR and with the operation with the HMD” - “I would like to recommend the system to other patients that have regular Lokomat^®^ sessions, because it is more challenging, motivational and beneficial for the rehabilitation”

Abbreviations: VR = Virtual Reality.

**Table 6:** Open-ended replies from the participants.

Q12—Please note 3 things you liked about using the Lokomat^®^ together with a VR headset
- simple to use - the reality of the environment was sufficient - time flies for the patients with its use - the whole installation is fairly easy to use - the patients had more motivation for training-especially those, who had several Lokomat^®^ trainings before participation - patients were able to have longer training sessions, which was seen in quite a few cases - I liked the advanced type of training with double tasking—walking and attention to the surroundings - this type of training arouses the patient’s interest and increases his participation
Q13—Please note 3 things about using the Lokomat^®^ together with a VR headset you would like to change
- would like to add more challenges during walking - to control the direction of walking according to the step length - possible more different environments, not just one scene, so that the patients could not be bored of walking in a single scene all the time - to change directions with legs’ performance - the system could be easier to use from the technological point of view, especially software in the HMD - the beneficial function of the Loko-VR training for the rehabilitation process would be the possibility to train particular walking features (swing phase, stance phase, gait symmetry, etc.) - it could be easier to access the VR application in the HMD - the HMD might be combined with the safety system in the case if the HMD falls from the patient’s head—however, there was no such case, but if the HMD would somehow fall to the treadmill, it could present an obstacle on the walking path

Abbreviations: Q = question; VR = virtual reality.

## Data Availability

The original contributions presented in the study are included in the article/supplementary material; further inquiries can be directed to the corresponding author/s.

## Supplementary Materials

The following supporting information can be downloaded at: https://www.mdpi.com/article/10.3390/brainsci14101002/s1, Video S1: Integration of immersive virtual reality into gait rehabilitation robotics (Loko-VR).
